# The 2nd European Carotid Surgery Trial (ECST-2): rationale and protocol for a randomised clinical trial comparing immediate revascularisation versus optimised medical therapy alone in patients with symptomatic and asymptomatic carotid stenosis at low to intermediate risk of stroke

**DOI:** 10.1186/s13063-022-06429-z

**Published:** 2022-07-27

**Authors:** Suk Fun Cheng, Twan J. van Velzen, John Gregson, Toby Richards, Hans Rolf Jäger, Robert Simister, M. Eline Kooi, Gert J. de Borst, Francesca B. Pizzini, Paul J. Nederkoorn, Martin M. Brown, Leo H. Bonati

**Affiliations:** 1 Stroke Research Centre, Department of Brain Repair and Rehabilitation, UCL Queen Square Institute of Neurology, University College London, London, UK; 2 Department of Neurology, Amsterdam UMC, University of Amsterdam, Amsterdam, The Netherlands; 3 Department of Medical Statistics, London School of Hygiene and Tropical Medicine, London, UK; 4 Faculty of Health and Medical Sciences, Surgery, University of Western Australia, Perth, Australia; 5 Neuroradiological Academic Unit, Department of Brain Repair and Rehabilitation, UCL Queen Square Institute of Neurology, University College London, London, UK; 6 Lysholm Department of Neuroradiology, National Hospital for Neurology and Neurosurgery, University College London Hospitals NHS Foundation Trust, London, UK; 7 Comprehensive Stroke Service, University College London Hospitals NHS Foundation Trust, London, UK; 8 Department of Radiology and Nuclear Medicine, CARIM School for Cardiovascular Diseases, Maastricht University, Maastricht, The Netherlands; 9 Department of Vascular Surgery, University Medical Center, Utrecht, The Netherlands; 10 Radiology, Department of Diagnostic and Public Health, University of Verona, Verona, Italy; 11 Department of Neurology, University Hospital Basel, Basel, Switzerland; 12 Department of Clinical Research, University of Basel, Basel, Switzerland; 13 Research Department, Reha Rheinfelden, Rheinfelden, Switzerland

**Keywords:** Carotid stenosis, Ischaemic stroke, Carotid endarterectomy, Carotid stenting, Risk prediction, Magnetic resonance imaging, Plaque imaging, Randomised controlled trial

## Abstract

**Background:**

Carotid endarterectomy is currently recommended for patients with recently symptomatic carotid stenosis ≥50%, based on randomised trials conducted 30 years ago. Several factors such as carotid plaque ulceration, age and associated comorbidities might influence the risk-benefit ratio of carotid revascularisation. A model developed in previous trials that calculates the future risk of stroke based on these features can be used to stratify patients into low, intermediate or high risk. Since the original trials, medical treatment has improved significantly. Our hypothesis is that patients with carotid stenosis ≥50% associated with a low to intermediate risk of stroke will not benefit from additional carotid revascularisation when treated with optimised medical therapy. We also hypothesise that prediction of future risk of stroke in individual patients with carotid stenosis can be improved using the results of magnetic resonance imaging (MRI) of the carotid plaque.

**Methods:**

Patients are randomised between immediate revascularisation plus OMT versus OMT alone. Suitable patients are those with asymptomatic or symptomatic carotid stenosis ≥50% with an estimated 5-year risk of stroke of <20%, as calculated using the Carotid Artery Risk score. MRI of the brain at baseline and during follow-up will be used as a blinded measure to assess the incidence of silent infarction and haemorrhage, while carotid plaque MRI at baseline will be used to investigate the hypotheses that plaque characteristics determine future stroke risk and help identify a subgroup of patients that will benefit from revascularisation. An initial analysis will be conducted after recruitment of 320 patients with baseline MRI and a minimum of 2 years of follow-up, to provide data to inform the design and sample size for a continuation or re-launch of the study. The primary outcome measure of this initial analysis is the combined 2-year rate of any clinically manifest stroke, new cerebral infarct on MRI, myocardial infarction or periprocedural death.

**Discussion:**

ECST-2 will provide new data on the efficacy of modern optimal medical therapy alone versus added carotid revascularisation in patients with carotid stenosis at low to intermediate risk of future stroke selected by individualised risk assessment. We anticipate that the results of baseline brain and carotid plaque MRI will provide data to improve the prediction of the risk of stroke and the effect of treatment in patients with carotid stenosis.

**Trial registration:**

ISRCTN registry ISRCTN97744893. Registered on 05 July 2012

## Administrative information

Note: the numbers in curly brackets in this protocol refer to SPIRIT checklist item numbers. The order of the items has been modified to group similar items (see http://www.equator-network.org/reporting-guidelines/spirit-2013-statement-defining-standard-protocol-itemsfor-clinical-trials/).

**Table:** 

**Title {1}**	**The 2**^**nd**^**European Carotid Surgery Trial (ECST-2): rationale and protocol for a randomised clinical trial comparing immediate revascularisation versus optimised medical therapy alone in patients with symptomatic and asymptomatic carotid stenosis at low to intermediate risk of stroke**
**Trial registration {2a and 2b}**	ISRCTN registry (ISRCTN97744893)
**Protocol version {3}**	Version 3.1
**Funding {4}**	National Institute for Health Research (NIHR) (grant number PB-PG-0609-19216), Stroke Association (grant number TSA 2013/04), Swiss National Science Foundation (grant number 32003B-156658), the Netherlands Organisation of Scientific Research (ZONMW; project nr. 843004107).
**Author details {5a}**	The first draft of the protocol was authored by the Chief Investigator, Martin M Brown with the assistance of the Trial Manager, Roland Featherstone (retired). Members of the Trial Steering Committee as listed on the trial website (www.ecst2.com), commented on the draft protocol and contributed to the final wording of the protocol.
**Name and contact information for the trial sponsor {5b}**	University College London (UK) UCL Queen Square Institute of Neurology Queen Square London WC1N 3BG United Kingdom
**Role of sponsor {5c}**	The sponsor played no part in the study design, data collection, management, analysis or interpretation of the data, writing of the protocol and the decision to submit the protocol for publication.

## Introduction

### Background and rationale {6a}

Atherosclerotic stenosis at or around the carotid bifurcation is associated with an increased risk of ischaemic stroke in the ipsilateral carotid artery territory and accounts for 10–20% of all transient ischaemic attacks (TIA) and ischaemic strokes [1]. The first European Carotid Surgery Trial (ECST) and the North American Carotid Endarterectomy Trial (NASCET) both showed a benefit of carotid endarterectomy (CEA) in preventing stroke in patients with symptomatic stenosis of ≥70% [2, 3]. These trials show that CEA carried a perioperative risk of stroke and death of around 7%. Moreover, CEA did not prevent all recurrent strokes. NASCET did not show any benefit of CEA in patients with <50% stenosis, but there was a small benefit in those with 50–69% stenosis [3]. The focus of guidelines based on ECST and NASCET has been to recommend CEA on the basis of the degree of carotid stenosis and this has dictated clinical practice to date. However, analysis of individual data from both these trials has shown that multiple factors in addition to the degree of stenosis, such as age, sex, time from index event, carotid plaque morphology and patient comorbidities, influenced the risk of future stroke in patients treated with medical therapy alone and also the amount of risk reduction through CEA [4].

**Fig. 1:**
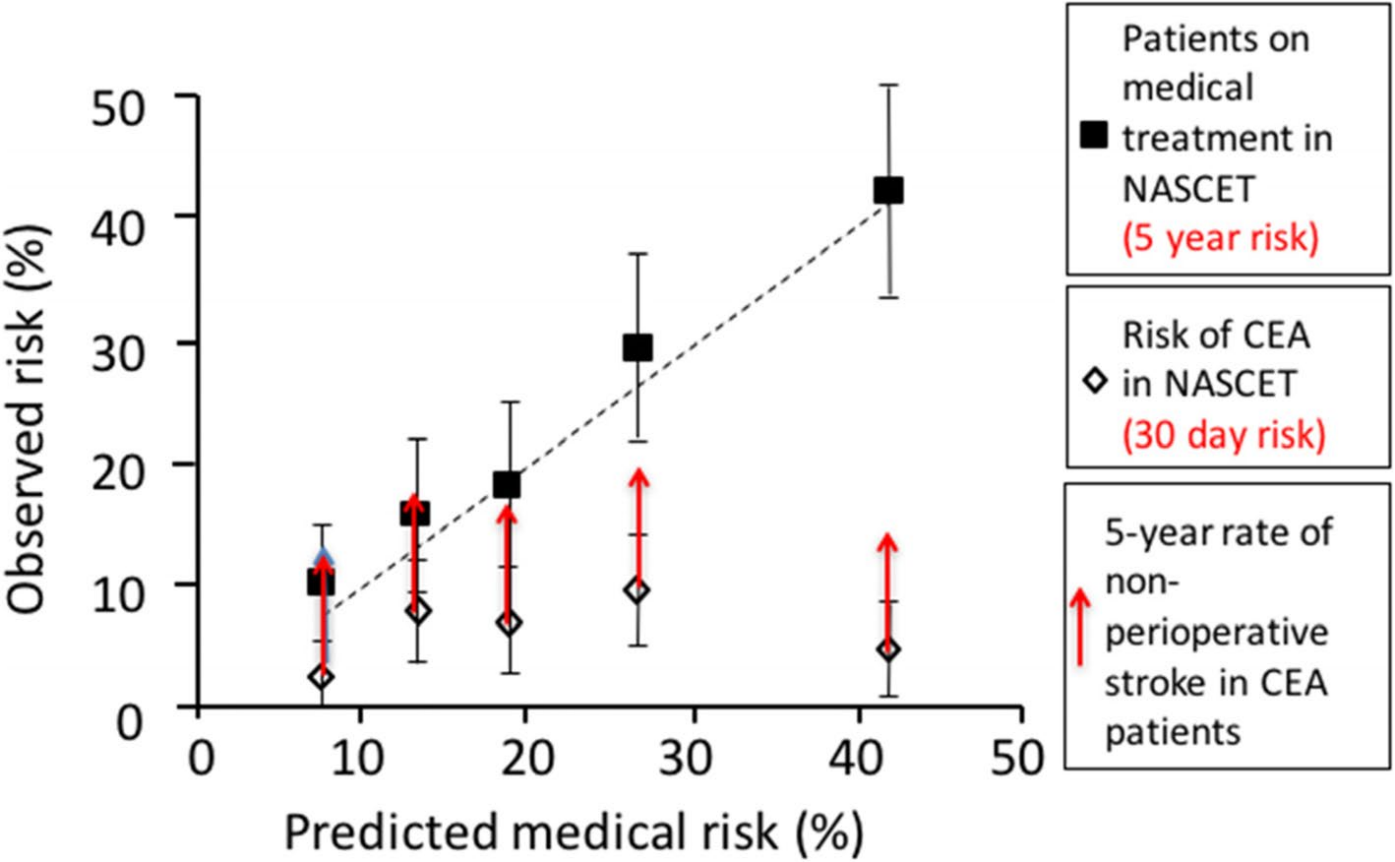
Figure showing reliability of a predictive model of 5-year risk of ipsilateral stroke derived from ECST data validated using results observed in NASCET. The figure is adapted from Rothwell et al., [4] [with the addition of arrows to show the 5-year risk of stroke in CEA patients]

A risk model based on these factors, derived from ECST and validated in NASCET showed that in patients with symptomatic carotid stenosis, the risk of ipsilateral stroke on medical therapy could be accurately predicted from baseline characteristics. It was evident from this analysis that only patients with a high risk of subsequent ipsilateral stroke when treated medically benefitted from CEA, while patients with a lower risk of stroke (5-year risk of <20%) did not benefit significantly because the benefit of surgery in the longer-term prevention of stroke did not justify the perioperative risk of stroke or death (Fig. 1) [4, 5].

In the Asymptomatic Carotid Surgery Trial (ACST), 3120 patients with asymptomatic carotid stenosis were randomised between immediate and deferred CEA [6, 7]. This trial did not include a fixed minimum stenosis percentage. The results of this trial showed a benefit of CEA in patients younger than 75 years of age with ≥60% carotid stenosis [6, 7]. Immediate CEA halved the 5-year stroke risk from 12 to 6% in these patients but the absolute risks were low (13.4% vs. 17.9%) with a net gain over 10 years of only 4.6% (95% CI 1.2–7.9). The median delay in this group was 1 month. The Asymptomatic Carotid Atherosclerosis Study (ACAS) showed a similar reduction in risk among patients with asymptomatic carotid stenosis ≥60% who received CEA versus medical therapy alone [8]. ACAS showed a 5-year stroke risk of 11.0% in the medical group compared to 5.1% in the surgical group with a relative reduction of stroke risk of 53% (95% CI 22–72). Carotid artery stenting (CAS) has emerged as an alternative method to revascularise carotid stenosis, but randomised trials in symptomatic patients suggested that the early risks of CAS outweigh those of CEA, except in patients younger than 70 years of age [9–11].

Previous trials of CEA versus medical therapy assessed only clinically manifest stroke reported by the patient and confirmed by adjudication in line with the definition of stroke as symptoms likely to be of cerebrovascular origin lasting at least 24 h. However, brain magnetic resonance imaging (MRI) is much more sensitive than clinical assessment in detecting ischaemic brain infarcts. Silent brain infarcts on MRI were found in 8% of participants in the Rotterdam study [12] and developed in 20% of participants after 10 years of follow-up in the Atherosclerosis Risk in Communities Brain Magnetic Resonance Imaging Study [13]. Silent infarcts on MRI are also seen after revascularisation procedures [14–17]. MRI can detect cerebral infarcts in the absence of clinical signs and symptoms and approximately doubles the number of episodes of cerebral infarction (clinically manifest and silent stroke) detected during follow-up. Thus, incorporation of new infarcts on brain MRI as an outcome measure is more sensitive than clinical assessment and therefore reduces the sample size required to demonstrate significant differences in the outcome between the two treatments. Another advantage of using MRI to assess outcome after stroke is that the determination of outcome is more objective than clinical assessment and outcome assessment can be done independently and blind to treatment and patient characteristics, unlike clinical assessment.

Furthermore, MRI is able to detect structural features of plaque instability, such as intra-plaque haemorrhage (IPH), which may improve the identification of patients benefitting from carotid revascularisation. The presence of IPH increases the risk of stroke in patients with carotid stenosis by a factor of more than ten and predicts risk independently of the degree of stenosis [18].

These considerations argued for a new trial to investigate the role of revascularisation in the setting of improved medical therapy adjusted to targets for risk factor control, i.e. optimised medical therapy (OMT), and using brain MRI to determine outcome measures. We therefore organised the Second European Carotid Surgery Trial (ECST-2).

### Objectives {7}

The objective of ECST-2 is to investigate the hypothesis that patients with carotid stenosis ≥50% associated with a low to intermediate risk of stroke will not benefit from additional carotid revascularisation when treated with optimised medical therapy. We also aim to investigate the hypothesis that the findings on baseline carotid plaque imaging can be used to predict future stroke risk and identify a subgroup of patients that will benefit from carotid revascularisation.

### Trial design {8}

ECST-2 is designed as a multicentre, prospective, randomised, controlled, open, multi-centre, non-inferiority clinical trial with blinded outcome adjudication of the primary outcome and 1:1 allocation to the two treatment arms balanced by minimisation.

## Methods: participants, interventions, and outcomes

### Study setting {9}

#### Participating centre requirements

Each centre is required to have a neurologist or physician with expertise in stroke to see patients prior to randomisation and to supervise follow-up and also a multidisciplinary process for ensuring that the management of individual patients with carotid stenosis is routinely discussed between physicians, surgeons, and radiologists. Centres are required to submit documentation demonstrating the training and experience of their investigators, together with an audit of the outcomes of carotid revascularisation at their centre. An expert credentialing committee is responsible for approving individual investigators and centre enrolment on the basis of acceptable outcomes from CEA, and also CAS if offered by the centre.

Thirty enrolled centres recruited and follow-up patients in the UK, Switzerland, the Netherlands, France, Germany, Italy and Canada. A list of the recruiting centres is available on the trial website (www.ecst2.com).

### Eligibility criteria {10}

The inclusion and main exclusion criteria for the trial are summarised in Table 1.

**Table 1:** Inclusion and exclusion criteria for ECST-2

**Inclusion criteria**	
• Patients over 18 years of age	
• Symptomatic or asymptomatic atherosclerotic carotid stenosis of at least 50% calculated using the NASCET criteria	
• Patients with a CAR score indicating a 5-year ipsilateral stroke risk of <20%	
• Patient is medically and neurologically stable and suitable for either CEA or CAS	
• Clinicians are uncertain about which treatment modality (OMT or OMT plus revascularisation) is best for the individual patient	
• Patient is able and willing to give informed consent	
**Exclusion criteria**	
• Patients with a modified Rankin score (mRS) > 2	
• Patients who are medically or neurologically unstable	
• Patients who have had coronary artery bypass grafting within 3 months or other major surgery within 6 weeks prior to randomisation	
• Patients with a CAR Score ≥20%	
• Occlusion of the carotid artery considered for randomisation	
• Patients not suitable for either surgery or stenting due to anatomical factors	
• Intraluminal thrombus within the carotid seen on ultrasound or angiography	
• Carotid stenosis caused by non-atherosclerotic disease, e.g. dissection, fibromuscular disease or neck radiotherapy	
• Previous CEA or CAS in the artery to be randomised	
• Recent revascularisation of the contralateral carotid artery or a vertebral artery or an intracranial artery carried out within 6 weeks prior to date of randomisation	
• Planned revascularisation of the contralateral carotid artery or a vertebral artery or an intracranial artery within 6 weeks	
• Patients who have a life expectancy of less than 2 years due to a pre-existing condition, e.g. cancer	
• Patients intolerant or allergic to all of the medications available for optimised medical therapy	

#### Use of the Carotid Artery Risk (CAR) score

Patients with asymptomatic or symptomatic carotid stenosis ≥50% with an estimated 5-year risk of stroke of <20% are identified by centres as suitable for inclusion in ECST-2 using the Carotid Artery Risk (CAR) score. The CAR score predicts the 5-year risk of ipsilateral stroke on the side of the stenotic carotid artery in patients treated with optimised medical therapy alone. The score was originally derived from the results of a Cox regression model using data from patients who had been randomised to medical treatment in the first ECST and was independently validated in the NASCET trial [2–5, 19]. This showed that the model produced accurate predictions of future risk of stroke on best medical therapy alone. Those with a high risk of stroke on medical therapy alone did not have any increase in their risk of perioperative stroke from carotid endarterectomy. The model has been widely used in routine clinical practice to select patients for carotid endarterectomy, both in its full web-based form published as the Carotid Stenosis Risk Prediction Tool by the Stroke Prevention Unit at the University of Oxford and in the form of the risk tables derived from the model [4]. However, given the evidence that the risks of stroke under medical therapy have declined since the time of ECST and NASCET, we recalibrated the model for use in ECST-2, taking into account the published effects of modern medical treatment of carotid stenosis and some additional risk data provided by the Stroke Prevention Unit. We named the output of the recalibrated model the Carotid Artery Risk (CAR) score. The CAR score is calculated based on the following patient characteristics: sex, age, diabetes, myocardial infarction, peripheral vascular disease, hypertension, percentage of carotid stenosis, the presence of a near carotid occlusion, plaque ulceration, time in days from the last ischaemic event to expected day of randomisation, and type of event (major stroke/multiple or single TIA/monocular event). These factors are used to provide an individualised estimate of the patient’s 5-year risk of ipsilateral stroke when treated with OMT alone. A tool is available on-line and as an app for use on smartphones and mobile devices to facilitate calculation of the CAR score in patients being considered for ECST-2.

The CAR score is used to select recently symptomatic patients at low or intermediate risk of stroke, which is defined as a 5-year risk of stroke ipsilateral to the target carotid artery calculated by the CAR score as <20%. Patients with a CAR score of ≥20% are excluded from the trial. Patients with asymptomatic stenosis or those with previous symptoms attributed to ipsilateral stenosis that had occurred more than 180 days prior to randomisation are also eligible for the trial and are assumed to have a low 5-year risk of ≤ 5% with OMT.

### Who will take informed consent? {26a}

Written informed consent from the participants is taken by the research investigator, who has been delegated to the task by the local principal investigator of the site. All investigators in the trial must have a valid Good Clinical Practice certificate.

### Additional consent provisions for collection and use of participant data and biological specimens {26b}

The consent form includes permission from participants for data, and samples of blood and tissue, to be retained for analysis and use in future studies.

### Interventions

#### Explanation for the choice of comparators {6b}

ECST and NASCET were completed over 20 years ago and since then medical therapy has improved significantly. The widespread use of statins, lower targets for blood pressure control and more effective and promptly started antithrombotic therapy have been shown to lower the incidence of recurrent stroke in prospective registries and trials of stroke prevention [20, 21]. There has been a fall in the proportion of smokers, reduction in baseline cholesterol and blood pressure levels and increased use of dual antiplatelet therapy after stroke or TIA [22, 23]. Subgroup analyses from randomised controlled trials and case-control studies have shown that statins lower the risk of stroke in patients with cerebrovascular disease by about a third and halve the numbers requiring CEA [24–27]. Systematic reviews have consistently shown a reduction in the average rates of ipsilateral stroke in patients with asymptomatic stenosis treated medically to as low as 1% per annum [28, 29]. There is also evidence that OMT with intensive risk factor control and adjustment of medical treatments to target, the annual risk of stroke associated with asymptomatic stenosis might be lowered to 0.6% per annum [30].

Coincidently with the improvement in medical management, there has also been a reduction in the operative risks of CEA since the time of the first trials, with most contemporary databases recording rates of perioperative stroke risk less than 2% in symptomatic patients [31–33]. Given the reductions in perioperative stroke risk and stroke risk with medical therapy alone since ECST, NASCET and ACST, the indications for carotid revascularisation are no longer clear. The data suggest that many patients in whom carotid revascularisation is recommended in line with guidelines based on the old trials may be receiving CEA without benefit and with a risk of harm. It is therefore very relevant to establish whether these patients should have OMT as the intervention of first-choice in the future.

#### Intervention description {11a}

The interventions being compared are immediate revascularisation with OMT versus OMT alone.

##### Optimised medical therapy (OMT)

OMT will be applied to both treatment groups immediately after randomisation (if not started beforehand) and includes antihypertensive medication and cholesterol-lowering medication in combination with a low cholesterol diet, adjusted to maintain pre-specified targets. The targets are specified by the local investigator for each patient in line with European, national or local guidelines. As a guide, we recommended a target total cholesterol of <4.0mmol/L (<155 mg/dL) and LDL cholesterol <2.0mmol/L (<77 mg/dL) and treatment to lower blood pressure to a target of 135/85 mmHg for patients aged <80 years and 150/90 mmHg for patients aged >80 years. Antiplatelet therapy is prescribed according to local guidelines. Anticoagulation is used as an alternative if indicated. Patients undergo targeted risk factor modification including smoking cessation and reduction of body weight if relevant.

##### Revascularisation

Patients allocated to revascularisation will have CEA (or in selected cases CAS, if considered preferable to CEA by local investigators) as soon as possible after randomisation and not later than 2 weeks for symptomatic patients or 4 weeks for asymptomatic patients, in addition to OMT. It is anticipated that the majority of patients allocated to revascularisation will be treated by endarterectomy. Detailed guidance is available to centres concerning relative and absolute contraindications to performing CAS in the trial.

#### Criteria for discontinuing or modifying allocated interventions {11b}

Cross-overs will be avoided unless clinically essential. Patient refusal of the treatment to which they are randomised will be minimised by careful consent. It is anticipated that early crossover will primarily occur in patients randomised to revascularisation in whom contraindications to intervention emerge after randomisation, so that they do not receive early revascularisation. Patients randomised to OMT alone should only receive revascularisation of the randomised artery if they have ipsilateral symptoms after randomisation which are attributed to the carotid stenosis and are considered to necessitate revascularisation. Such patients are not considered cross-overs. Patients requiring revascularisation because of new symptoms after allocation to OMT alone, as well as those requiring revascularisation of the contralateral, non-randomised carotid artery or needing a second carotid revascularisation procedure (e.g. because of symptomatic restenosis) can be re-treated with whichever method of revascularisation the local investigator considers most appropriate.

#### Strategies to improve adherence to interventions {11c}

Success at achieving risk factor control and treatment targets for OMT is monitored at follow-up. Investigators, patients and their general practitioner receive written advice concerning treatment targets. All patients are requested to schedule follow-up appointments depending on their availability to prevent redundancy of participants. The research team reminds the patient regarding their upcoming appointments through the mail, text or phone call.

#### Relevant concomitant care permitted or prohibited during the trial {11d}

Any concomitant care for any other illnesses is allowed throughout the study.

#### Provisions for post-trial care {30}

After completion of follow-up, patients will continue to receive optimised medical therapy according to local practice.

### Outcomes {12}

The primary outcome measure is stroke in any territory at any time, or periprocedural death attributed to carotid revascularisation. The primary analyses will examine the following question: What is the difference in the long-term survival free of any stroke, or periprocedural death in patients with atherosclerotic carotid stenosis at lower risk for stroke after randomisation to a policy of carotid revascularisation with OMT compared to OMT alone?

The primary outcome measure for the initial MRI-based analysis of the trial is the combined 2-year rate of any clinically manifest stroke, new cerebral infarct on MRI, myocardial infarction or periprocedural death. Secondary outcome measures include hyperperfusion syndrome, new-onset epileptic seizure, any hospitalisation for vascular disease, carotid revascularisation during follow-up other than that allocated at randomisation, cranial nerve palsy attributed to revascularisation, haematoma caused by treatment requiring surgery, transfusion or prolonging hospital stay, other adverse events attributed to medical treatment or revascularisation, stenosis progression (defined as recurrent stenosis of the randomised artery after revascularisation, or progression in severity of stenosis in a non-revascularised artery), further revascularisation of the randomised artery after the initial attempt, decline in cognitive function assessed by the Montreal Cognitive Assessment (MoCA) and decline in functional status as assessed by an increase in the modified Rankin score (mRS). In addition, measures will be reported relating to the quality of life and health status, health service use (e.g. length of stay in hospital, surgery, medications) and health service costs.

### Imaging protocols

#### Brain MRI

Mandatory MRI sequences include fluid-attenuated inversion recovery (FLAIR), to assess chronic ischaemic brain infarcts and markers of small vessel disease; diffusion-weighted imaging (DWI) to assess acute ischaemic brain lesions; and susceptibility-weighted imaging (SWI) or, if not available, T2*-weighted gradient-echo imaging (GRE), to detect cerebral micro-haemorrhages and other haemorrhagic brain lesions (Table 2). Centres are free to use magnetic field strengths of either 1.5 or 3.0 Tesla but should use the same scanner for each trial patient.

**Table 2:** MR imaging protocols in ECST-2

**Brain MR imaging**		
Required sequences	T1-weighted imaging T2-weighted imaging Fluid-attenuated inversion recovery (FLAIR) Diffusion-weighted imaging (DWI) Gradient echo T2*-weighted imaging (or susceptibility-weighted imaging [SWI])	
**Carotid plaque MR imaging**		
Required sequences	3D time of flight (TOF) 3D magnetisation-prepared rapid gradient-echo (MPRAGE) or an equivalent sequence that allows identification of intraplaque haemorrhage	
Optional sequences^a^	3D with contrast	Pre- and post-contrast T1-weighted SPACE/CUBE/VISTA imaging
	2D with contrast	Pre- and post-contrast black blood T1-weighted imaging
	3D without contrast	T1-weighted and T2-weighted black blood SPACE/CUBE/VISTA imaging
	2D without contrast	Black blood T1-weighted imaging T2-weighted imaging

^a^The option is given to use intravenous gadolinium contrast. It is preferred to use 3D imaging however there is an option for 2D imaging

#### Carotid plaque imaging

MRI is far superior to computed tomography (CT) in identifying the different components of a carotid plaque, in particular IPH. Several carotid plaque MRI protocols are summarised in a recent white paper [34]. Centres are given flexibility to choose their preferred plaque imaging protocol according to their local expertise from within a limited number of sequences varying in complexity and performed with or without the use of gadolinium-based contrast medium (Table 2). The basic protocol, which is well suited for demonstrating IPH, requires two sequences: 3D time of flight (TOF) and 3D magnetisation-prepared rapid gradient-echo (MPRAGE) or an equivalent MRI sequence that allows visualisation of IPH. This minimum requirement could be implemented at any centre using a standard head-and-neck coil on a 1.5 or 3.0 Tesla system, although the latter is preferable. The sequences of the basic protocol will allow quantification of stenosis, as well as detection of plaque ulceration, calcification and IPH [34]. Ulcerations will be scored on contrast-enhanced MR angiography, alternatively on 3D TOF, or, if unavailable, any other sequence that allows identification of plaque ulceration. Plaque ulceration is defined as an intimal defect larger than 1 mm in width [35]. IPH is identified as a hyperintense region (compared to the sternocleidomastoid muscle) in the bulk of the plaque on MPRAGE or if unavailable, TOF images or any other sequence that allows identification of IPH [34, 36].

More advanced sequences that have been designed to image plaque burden, the status of the fibrous cap and the size of lipid-rich necrotic core, based on recent expert consensus recommendations, are optional [34]. A thin or ruptured fibrous cap is defined as an interrupted region of signal enhancement adjacent to the lumen on the post-contrast images overlying the lipid-rich necrotic or full absence of a region with signal enhancement adjacent to the lumen overlying the lipid-rich necrotic core [34, 37]. A quality score is used to indicate the certainty on the score. A lipid-rich necrotic core is identified as an area in the bulk of the plaque that is hyperintense on the T1-weighted black blood MR images, does not enhance on the post-contrast T1-weighted black blood images, and potentially is hypointense in the same region on T2-weighted MR images.

#### Duplex ultrasound

Carotid duplex ultrasound will be performed at baseline and annually to estimate the percentage diameter reduction and grading of stenosis of the carotid artery on the randomised side and on the contralateral side. The degree of stenosis will be determined in the central trial office based on the peak systolic velocities of the common carotid and the internal carotid arteries and the end diastolic velocity of the internal carotid artery, on the basis of predefined, standardised flow velocity criteria, which equate well with the severity of carotid stenosis measured on catheter angiography using the NASCET method [38, 39].

### Participant timeline {13}

Table 3 summarises the timing of baseline and follow-up visits and investigations given in the protocol. Prior to randomisation, the following investigations are required: routine serum investigations, electroca rdiogram, imaging of both carotid bifurcations showing the severity of stenosis bilaterally and brain MRI. If MR is contra-indicated or not available within a reasonable time period for any reason, brain CT should be done instead. Baseline data will be collected at randomisation and includes the mRS assessed by using the Rankin Focused Assessment (RFA). Patients also complete the European Quality-of-Life 5 Dimension Questionnaire (EQ-5D) [40] at baseline and the MoCA [41]. Patients are followed up by a neurologist or stroke physician, or a clinician/research practitioner under the close supervision of a neurologist or stroke physician. Required investigations during follow-up include at least annual measurements of the blood pressure and serum cholesterol and an annual carotid duplex ultrasound. Brain MRI is required at the time of randomisation and at 2 and 5 years after randomisation to determine the rate of the MR-based outcome events, unless contra-indicated. Additional brain MRI is required whenever a suspected stroke or a TIA occurs during the study period to confirm the clinical diagnosis. In patients with contraindications for MRI or if MRI was not available within a reasonable period, brain CT is allowed instead. MRI of the carotid plaque is also done prior to randomisation at centres able to perform the required sequences.

**Table 3:** Outline of baseline and follow-up appointments including investigations required and target time window

Visit/follow-up	Investigations	Target time window
Baseline	Brain MRI	14 days in symptomatic stenosis or 28 days in asymptomatic stenosis before or after randomisation, done before any revascularisation procedure
	Initial carotid imaging	120 days before randomisation up to the day of randomisation
	Confirmatory second carotid imaging	14 days before randomisation up to the day of randomisation
	Clinical assessment, blood pressure Baseline blood lipids and glucose, serum troponin levels ECG MoCA, EQ-5D, RFA	14 days before randomisation up to the day of randomisation
Post-procedure visit in revascularised patients only	Clinical assessment, blood pressure Troponin and ECG	Day of treatment +48 h ±24 h
4–6 weeks post-randomisation	Clinical assessment, blood pressure MoCA, EQ-5D, RFA	Day of treatment +30 days ±7 days in revascularised patients
	ECG and troponin in the OMT arm	Day of randomisation +42 days ±7 days in OMT only patients
	Carotid ultrasound in revascularised patients	Day of treatment +30 days ±7 days
	Brain MRI (optional)	Day of treatment +30 days ±7 days in revascularised patients Day of randomisation +42 days ±7days in OMT only patients
3 months post-randomisation	Telephone follow-up	Day of randomisation +90 days ±14 days
6 months post-randomisation	Clinical assessment, blood pressure EQ-5D, RFA Fasting lipids and glucose	Day of randomisation +180 days ±14 days
Annual follow-up	Clinical assessment, blood pressure EQ-5D, RFA Fasting lipids and glucose Carotid ultrasound MoCA and brain MRI at 2 and 5 years	Day of randomisation +X years ±1 month
Bi-annual telephone follow-up	Telephone follow-ups between annual follow-ups	Day of randomisation +X years +6 months ±1 month

### Sample size {14}

We expect a rate of stroke in the OMT arm of approximately 3% at 2 years after randomisation. Silent ischaemic infarcts on MRI seem to occur at twice the frequency of clinically manifest strokes [42, 43]. Thus, we expect about 9% of patients on OMT to experience clinically manifest stroke or have silent new ischaemic infarct on MRI after 2 years of follow-up. In those allocated to revascularisation, we expect a peri-operative risk of stroke of 3% and a subsequent 2-year risk of stroke of 2%. Thus, we expect about 15% of patients in the revascularisation arm to experience clinically manifest stroke or have silent new ischaemic infarct on MRI after 2 years of follow-up.

The rate of clinically manifest periprocedural myocardial infarction (MI) in carotid revascularisation is about 1% [44]. The 2-year risk of non-periprocedural MI is expected to be about 2% using the Framingham model [45] based on the characteristics of patients in the International Carotid Stenting Study (ICSS) [46]. We therefore estimated a total 2-year risk of MI of 3% in patients randomised to revascularisation and 2% in those randomised to OMT. The periprocedural death rate (unrelated to MI or stroke) in ICSS patients undergoing CEA was 0.1%. The rate of the primary outcome event in ECST-2 of clinically manifest stroke, new ischaemic infarct on MRI, MI or periprocedural death at 2 years was therefore estimated at 11% in the OMT arm alone and 18% in the revascularisation arm. Assuming these outcome event rates, 314 patients (157 in each arm) provide 80% power to demonstrate that OMT is non-inferior to OMT plus revascularisation with a non-inferiority margin of 4%. Allowing for a dropout of 15% of patients missing their 2-year MRI, we plan to recruit 360 patients for the MRI-based analysis.

We originally calculated a sample size of 2000 patients for the full ECST-2 trial, details of which can be found in the trial protocol. The sample size calculation for the continuation or re-launch of the study will be based on the outcome rates determined in the initial analysis.

### Recruitment {15}

The strategies adopted to achieve adequate participant enrolment included consultation with collaborators at investigators’ meetings and regular communication with investigators via a regular newsletter. Centres are also provided with on-site or remote training at set up of the centre or when a new investigator joined the team. Centres are provided with individualised feedback on the progress with recruitment and reminders concerning patient follow-up and missing data. All investigators have email access to a trial manager based in the central office and to the chief investigator, who answers queries promptly, including those related to participant enrolment.

ECST-2 allows centres to use their normal clinical practice to identify patients with carotid stenosis. The NASCET criteria were specified for measurement of the degree of carotid stenosis [38, 47]. The imaging modality to grade carotid stenosis and assess suitability for revascularisation is not specified, but confirmation using a second imaging modality before randomisation is required.

### Assignment of interventions: allocation

#### Sequence generation {16a}

A randomisation tool is provided to investigators on a secure password-protected website (www.sealedenvelope.com). Patients are randomly allocated in a 1:1 ratio to be treated either by (1) immediate carotid revascularisation plus OMT or (2) by OMT alone.

### Concealment mechanism {16b}

Allocation is balanced by minimisation on the following factors: recruitment centre, planned method of revascularisation (endarterectomy, stenting, angioplasty) and risk group (asymptomatic with stenosis ≥ 70%, asymptomatic with stenosis < 70%, and CAR score in symptomatic stenosis). The minimisation incorporates a random component so that the treatment group that minimises imbalance is chosen with probability of 0.85. Separate randomisation lists are maintained according to whether endarterectomy or stenting was the pre-specified treatment.

### Implementation {16c}

A study number is assigned at the point of enrolment after the patient signed and dated the informed consent. The investigator or research team assigns the patient to the intervention according to the intervention allocated to the patient.

### Assignment of interventions: blinding

#### Who will be blinded {17a}

Because of the nature of the interventions being compared it is not practical to blind patients or clinicians to the treatment allocated. However, all follow-ups will be performed by neurologists or stroke physicians or staff under their close supervision rather than the surgeons or interventionists performing revascularisation. The central office staff, chief investigator and Steering Committee will all remain blinded to the cumulative event rate in the two arms until the interim analysis is complete. Follow-up MRI and CT scans will be analysed blind to treatment received, providing a non-biased comparison of outcome in the two groups. Major outcomes will be adjudicated by an adjudication committee who is blinded to the allocated treatment.

#### Procedure for unblinding if needed {17b}

The central office staff, chief investigator and Steering Committee will remain blinded until the completion of the first analysis of the trial results, unless advised to the contrary by the Data Monitoring Committee.

### Data collection and management

#### Plans for assessment and collection of outcomes {18a}

Data will be collected and captured using an electronic case record form on Sealed Envelope (www.sealedenvelope.com). All 30-day post-procedural complications after revascularisation and other outcome events will be reported in detail to the central office. At each visit, levels of disability will be assessed using a structured interview to determine the mRS and any outcome events notified to the Central Office. In patients with suspected or confirmed TIA or stroke during follow-up, an additional MRI brain scan will be done to confirm the diagnosis (unless contraindicated, when a CT will be done instead) together with an ultrasound or other re-examination of the carotid arteries. Copies of the images and reports will be returned to the trial office to assist with central adjudication and analysis.

#### Plans to promote participant retention and complete follow-up {18b}

Treatment refusals and cross-overs are minimised by careful informed consent. The effectiveness of the OMT regime will be monitored at follow-up; GPs are asked to monitor patients’ blood pressure and cholesterol at regular intervals and these data will be collected at the scheduled trial follow-up visits.

#### Data management {19}

Digital copies of the MRI or CT scans and the imaging of the carotid bifurcation will be sent to the Central Trial Office for central analysis together with copies of ECGs and the written reports of the imaging studies. Where more than one imaging modality has been used to image the brain or carotid bifurcations, copies of all the investigations will be sent to the Central Office. All brain and carotid images are stored on a central secure database and will be analysed by expert trial neuroradiologists and/or trained observers to refine prediction of risk and benefit of carotid revascularisation.

#### Confidentiality {27}

All data obtained in this trial is kept and handled in a confidential manner in accordance with applicable laws and regulations. Sealed envelope servers follow the latest cipher suites and security guidelines and all web-based traffic in transit is encrypted.

#### Plans for collection, laboratory evaluation and storage of biological specimens for genetic or molecular analysis in this trial/future use {33}

Blood samples for genetic analysis are collected and stored in a secure location for future analysis.

### Statistical methods

#### Statistical methods for primary and secondary outcomes {20a}

The primary analysis will be by intention-to-treat; i.e. patients will be analysed by their randomly allocated group despite what treatment they actually receive. A per-protocol analysis will be performed as an indicator of the actual treatment effect, excluding (i) patients randomised in the OMT group who undergo revascularisation within 6 weeks of randomisation without prior symptoms, as well as (ii) patients randomised in the revascularisation group who do not undergo revascularisation within 6 weeks of randomisation.

#### Interim analyses {21b}

An initial analysis will be performed to assess the safety of the treatment policies and inform the design and sample size calculations for the full trial, with the combined 2-year outcome of any stroke, new cerebral infarct on MRI, MI or periprocedural death. During the period of recruitment to the study, interim analyses of mortality and of any other information that is available on major endpoints (including serious adverse events believed to be due to treatment) will be provided, in strict confidence, to the Data Monitoring Committee by the trial statistician, along with any other analyses that the Committee may request. In the light of these analyses, the Data Monitoring Committee will advise the chairman of the Steering Committee if, in their view, the randomised comparisons in ECST-2 have provided both (i) “proof beyond reasonable doubt” that for all, or for some, specific types of patients, one particular treatment is clearly indicated or clearly contraindicated in terms of a net difference in outcome, and (ii) evidence that might reasonably be expected to materially influence patient management. Appropriate criteria of proof beyond reasonable doubt cannot be specified precisely, but a difference of at least three standard deviations in an interim analysis of a major endpoint may be needed to justify halting, or modifying, the study prematurely.

#### Methods for additional analyses (e.g. subgroup analyses) {20b}

Subgroup analyses will examine the influence of individual risk factors for outcome events and potential modifiers of treatment effect. Subgroups of particular interest will be age (dichotomised at the mean and as a continuous variable), sex, symptomatic versus asymptomatic carotid stenosis, CAR score, diabetes, hypertension, severity of stenosis, contralateral stenosis or occlusion, type of most recent event, multiple symptoms, centre recruitment and time from event to revascularisation. In addition, subgroup analyses according to the presence or absence of IPH will be done. The results will also be analysed according to adherence to OMT targets.

#### Methods in analysis to handle protocol non-adherence and any statistical methods to handle missing data {20c}

Patients who stop attending regular follow-up visits without occurrence of an outcome event will be censored at the time of their last visit. Censoring is non-informative. The trial management team strives for a high level of data completeness. Statistical imputation of missing data is not foreseen.

### Oversight and monitoring

#### Composition of the coordinating centre and trial steering committee {5d}

The study will be organised on behalf of the collaborators by the UCL Queen Square Institute of Neurology in London. The office will be responsible for protocol design, data collection and management and analysis of the results in consultation with the Steering and Data Monitoring Committees. The Steering Committee consists of the chief investigator and individuals participating in and independent of the trial with experience in vascular neurology, cardiovascular disease, vascular surgery, vascular radiology, interventional neuroradiology, health economics, clinical trials and statistics, together with patient and carer representatives. Individual Country Coordinators from the most active centres will be represented on the committee. The Steering Committee will have an independent Chairman and will oversee the overall management of the trial.

#### Composition of the data monitoring committee, its role and reporting structure {21a}

The safety aspects of the trial will be overseen by an independent Data Monitoring Committee with expertise in neurology, clinical trials, medical statistics, vascular surgery and clinical pharmacology.

#### Adverse event reporting and harms {22}

Outcome events will be documented in detail by the investigating centre. Patients suffering from a stroke should have an MRI brain scan as soon as possible after the event (or CT if contraindicated). An electronic copy of this will be submitted together with a report of the event. For MI, documentation of changes in cardiac biomarkers and copies of ECG recordings should be returned to the trial office. The event report will include copies of discharge summaries, death certificates and post-mortem results if relevant. Deaths of UK patients will be tracked by flagging patients against the UK Registry of Births and Deaths. Disability after stroke and cranial nerve palsy will be assessed 30 days and 6 months after treatment or onset, using the mRS. Duration of symptoms will be recorded and outcome events will be classified as disabling if the mRS is 3 or more. Reports of outcome events will be censored after receipt at the central office to remove information concerning treatment allocation as far as practical and then sent for adjudication as soon as sufficient information concerning the event has been received. Major outcome events will be adjudicated by two neurologists or cardiologists, depending on the reported event, at least one of whom will be independent of the trial. If the two physicians differ significantly in the classification of the event, the data will be sent to another independent adjudicator for their views. Major conflicts will be resolved by consensus or a majority view if consensus is not achieved. Additional information may be requested at any time by the central office or an adjudicating physician.

If the local investigator or other member of the team, at a trial centre, has concern about the outcome of their trial procedures, they should inform the trial office, which will organise a blinded assessment of the relevant outcome events. This will be submitted by the central office to the chairman of the Data Monitoring Committee who may recommend further action, such as suspending randomisation at the centre. Similarly, the database manager at the trial office will monitor outcome events and if there are two consecutive deaths or three consecutive major events at a single centre within 30 days of treatment in the same arm of the study, then assessment of the events will be triggered. A cumulative major event or death rate of 10% or more over 20 cases would also trigger careful assessment of the relevant outcome events.

#### Frequency and plans for auditing trial conduct {23}

The progress of the study is assessed at regular intervals determined by the Data Monitoring Committee.

#### Plans for communicating important protocol amendments to relevant parties (e.g. trial participants, ethical committees) {25}

The Central Trial Office consults with the collaborators at investigators’ meetings and at other times as necessary. Communication with investigators also takes place via a regular newsletter and the trial website.

#### Access to trial data {29}

Non-identifiable data and materials from the final trial dataset will be freely available to any suitable individual or organisation in response to a reasonable and well-motivated request addressed to the chief investigator. Research outputs arising from any analysis of trial data should appropriately acknowledge the funding and support received for the research.

#### Dissemination plans {31a}

The main results of the trial will be submitted for presentation at relevant scientific conferences. Publications resulting from the analysis of the trial will be disseminated through peer-reviewed publications. Key publications will be made available to the public through open-access publication.

#### Authorship eligibility {31b}

 A writing committee appointed by the chief investigator will prepare the main manuscripts on behalf of the trial investigators. The writing committee will include representatives from the trial office staff, trial steering committee and the most active recruiting centres. Members of the writing committee will be expected to conform with the requirements for authorship and contribution published by the International Committee of Medical Journal Editors.

#### Plans to give access to the full protocol, participant-level data and statistical code {31c}

The full trial protocol (version 3.1) is available to download from the trial website (www.ecst2.com). It is not planned that the participant-level dataset and statistical code will be made publicly available, except as outlined under the heading ‘Access to trial data’ above.

## Discussion

The objective of ECST-2 is to test the hypothesis that patients with carotid stenosis ≥50% associated with a low to intermediate risk of stroke will not benefit from additional carotid revascularisation when treated with optimised medical therapy, because vascularisation surgery will cause more periprocedural vascular events than are prevented by removal of the plaque. ECST-2 is designed to closely reflect current medical practice so that the results of the trial are applicable to everyday medicine.

ECST-2 differs from previous and ongoing trials of carotid stenosis in several important aspects. Firstly, to select patients for the trial, rather than selecting patients on the basis of symptom status as in previous carotid trials, ECST-2 uses an individualised risk assessment tool, the Carotid Artery Risk (CAR) score, which is derived from a validated model based on several clinical variables. It is notable that many patients with symptomatic stenosis have a low predicted risk of future stroke similar to that of patients with asymptomatic stenosis. Therefore, ECST-2 includes both patients with lower risk symptomatic stenosis and those with asymptomatic stenosis, viewing risk as a spectrum for which symptomatic status contributes only a part. Secondly, ECST-2 defined OMT using international guidelines and encouraged achievement of these guideline-based treatment targets, unlike previous carotid trials which did not define best medical treatment. Thirdly, ECST-2 routinely uses MRI as an additional, sensitive and objective tool that can be analysed blind to treatment to assess ischaemic or haemorrhagic brain injury at enrolment and occurring as a consequence of revascularisation or the failure of medical treatment during follow-up. Finally, the trial also represents a unique opportunity to investigate whether the findings on MRI of the carotid plaque help select patients who benefit from revascularisation. Plaque MRI has been assessed in non-randomised prospective carotid disease studies, which suggest that certain features including IPH, visualised using appropriate MR sequences, predict a high rate of future ischaemic stroke on medical therapy alone, independent of the degree of stenosis [18]. The presence of IPH correlates with a higher risk of ipsilateral stroke in patients with symptomatic stenosis with an unadjusted hazard ratio of 10.2 (95% CI 4.6–22.5) [18, 48, 49]. However, these studies have not established whether IPH also predicts a higher rate of perioperative stroke in patients receiving early revascularisation, nor whether patients with IPH, who otherwise appear to have a low risk of recurrent stroke and randomised to OMT only, benefit from carotid revascularisation. ECST-2 provides a unique opportunity to answer these questions.

We recognise that event rates might be different to those predicted and therefore the analysis of the trial might not yield sufficient evidence to prove or refute our hypothesis. Thus, to determine the optimum management of low-risk patients with carotid stenosis, the results of further trials will be needed, including those of CREST-2 (Second Carotid Revascularization Endarterectomy versus Stenting Trial), SPACE-2 (Stent Protected Angioplasty versus Carotid Endarterectomy), and ACTRIS (Endarterectomy Combined With OMT vs OMT Alone in Patients With Asymptomatic Severe Atherosclerotic Carotid Artery Stenosis at Higher-Than-Average Risk of Ipsilateral Stroke, https://clinicaltrials.gov/ct2/show/NCT02841098) [50, 51].

Current guidelines are based on the results of randomised clinical trials performed several decades ago, despite remarkable falls in rates of stroke associated with carotid stenosis related to improvements in medical and surgical practice. The results of ECST-2 and other ongoing trials will allow the guidelines to be updated with more current figures for surgical complication rates and risks on OMT alone.

A further main aim of ECST-2 is to determine whether the use of the CAR score, baseline brain MRI and baseline plaque MRI can be used to improve risk prediction in individual patients. We anticipate that the results of ECST-2 together with the results of other studies of brain and plaque imaging will usher in an era of personalised stroke prevention for patients with atherosclerotic carotid artery stenosis in which treatment (OMT with or without revascularisation) is recommended for patients based on individualised assessment of their future stroke risk incorporating imaging-based analysis (Table 3).

## Trial status

ECST-2 was registered as an international clinical trial in the ISRCTN registry in July 2012 (ISRCTN97744893). It was originally conceived that an initial analysis would be performed, based on the MRI analysis of cerebral infarction and haemorrhage, as described above, to inform the design and sample size of a continuation of the trial, with an anticipated final sample size of 2000 required to perform an analysis based on clinically evident events only. However, the technology available to assess risk was rapidly advancing with the development of MRI of the carotid plaque and the demonstration that IPH appeared to be a powerful predictor of stroke outcome in lower-risk patients treated medically. The steering committee therefore decided to suspend randomisation in ECST-2 in October 2020 after recruiting sufficient patients to conduct both the planned brain imaging-based analysis of outcome events and the plaque MRI analysis. The COVID-19 pandemic in 2020 and 2021 impacted on the follow-up of the study population by delaying visits at some centres, but currently, follow-up is planned to continue to allow a 5-year follow-up of the last enrolled patients. A decision will be made on the design for the re-launch of randomisation in ECST-2 with a similar or altered design when the results of the initial analysis are available. The steering committee was also cognisant that new funding would be required to re-launch recruitment in the trial.

## Abbreviations

ACST: Asymptomatic Carotid Surgery Trial
CAR: Carotid Artery Risk
CAS: Carotid artery stenting
CEA: Carotid endarterectomy
CREST-2: Second Carotid Revascularization Endarterectomy versus Stenting Trial
CT: Computed tomography
DWI: Diffusion-weighted imaging
ECST: European Carotid Surgery Trial
ECST-2: Second European Carotid Surgery Trial
EQ-5D: European Quality-of-Life 5 Dimension Questionnaire
FLAIR: Fluid-attenuated inversion recovery
GRE: Gradient-echo imaging
ICSS: International Carotid Stenting Study
IPH: Intra-plaque haemorrhage
MI: Myocardial infarction
MoCA: Montreal Cognitive Assessment
MPRAGE: Magnetisation-prepared rapid gradient-echo
MRI: Magnetic resonance imaging
mRS: Modified Rankin score
NASCET: North American Carotid Endarterectomy Trial
OMT: Optimised medical therapy
RFA: Rankin Focused Assessment
SWI: Susceptibility-weighted imaging
TIA: Transient ischaemic attack
TOF: Time of flight
UCL: University College London
